# Supplementary material for: The viral protein corona directs viral pathogenesis and amyloid aggregation
Source: Nat Commun. 2019 May 27;10:2331. doi: 10.1038/s41467-019-10192-2 (PMC6536551; doi:10.1038/s41467-019-10192-2)
Supplement: Supplementary file 1 — Supplementary Information [file 41467_2019_10192_MOESM1_ESM.pdf]

Supplementary Information

The Viral Protein Corona Directs Viral Pathogenesis and Amyloid Aggregation

Ezzat K. et al.

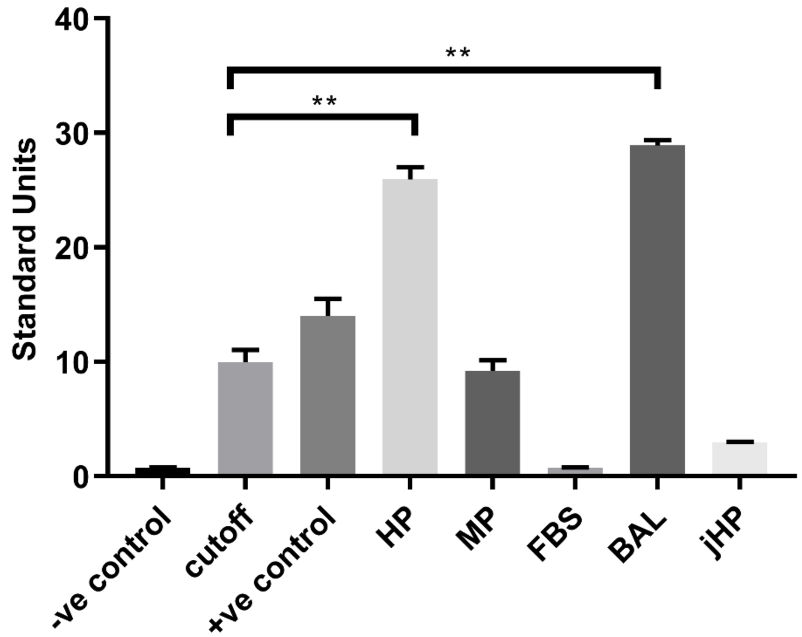

Supplementary Figure 1. ELISA analysis of anti-RSV IgG antibodies in different biological fluids. Different biological fluids were diluted to a protein concentration of 0.3 mg/ml, and then ELISA was used to quantify RSV specific IgG antibodies. Significant differences were assessed by Mann-Whitney test and are indicated by $^{**}P$ <.01. Data are shown as means + SEM of six replicates from two separate experiments.

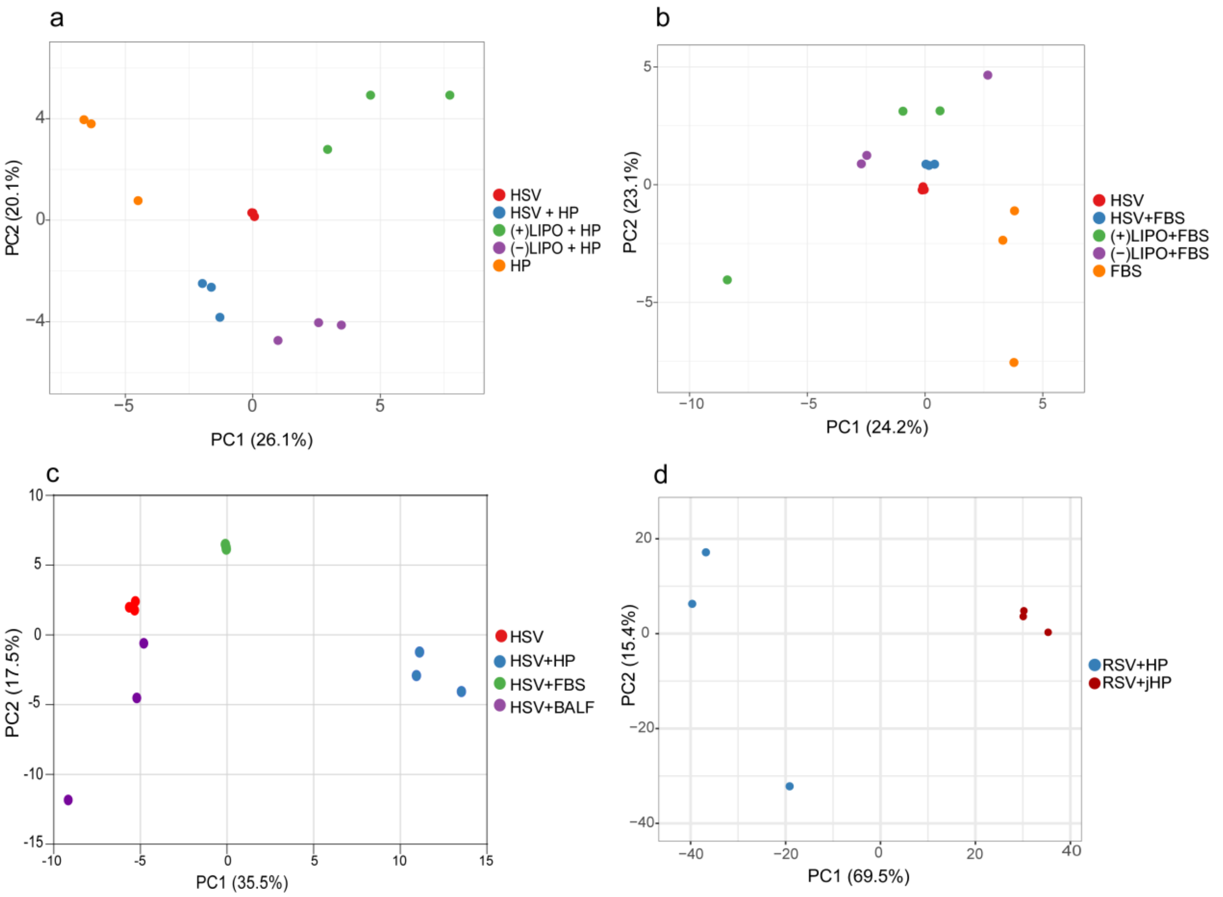

Supplementary Figure 2. Principal component analyses (PCA) of the corona proteomic profiles. Triplicate samples were incubated with 10% solutions of different biological fluids for 1h at 37 ˚C, then re-harvested, washed and finally analyzed by MS. (-)Lipo = negatively charged lipid vesicles, 200 nm, (+)Lipo = positively charged lipid vesicles, 200 nm. Only proteins significantly detected (FDR 1%) in all three replicates in each condition were used. (**a**) PCA comparing proteomic profiles of HSV-1 and controls in human plasma (HP) (**b**) PCA comparing proteomic profiles of HSV-1 and controls in fetal bovine serum (FBS). (**c**) PCA comparing proteomic profiles of HSV-1 in different biological fluids. (**d**) PCA comparing corona proteomic profiles of RSV in adult human plasma (HP) vs. juvenile human plasma (jHP).

a

b

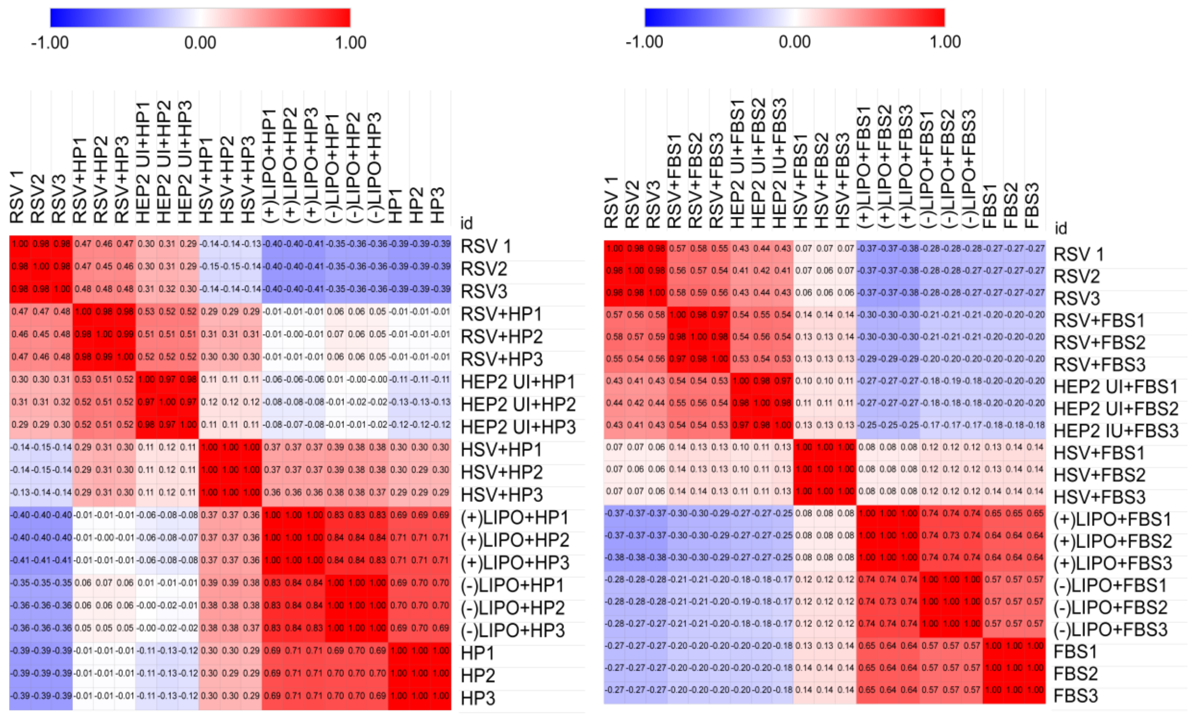

c

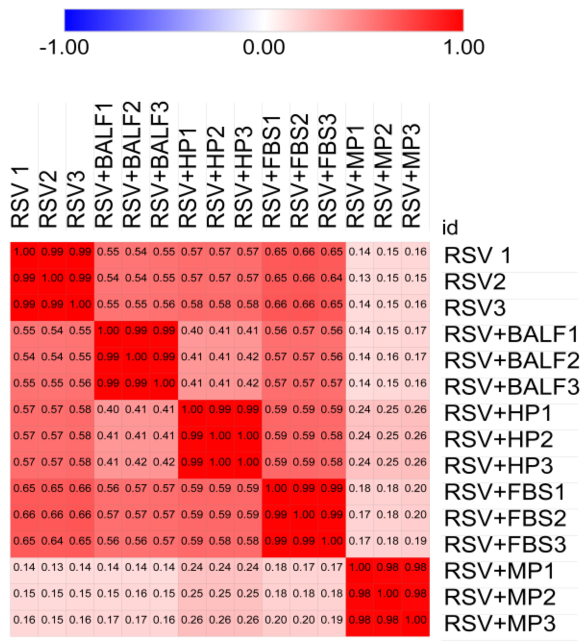

d

MATRIX	CV%
FBS	29
HP	19
(-)Lipo + FBS	42
(-)Lipo + HP	36
(+)Lipo + FBS	41
(+)Lipo + HP	28
HSV+FBS	16
HSV+HP	23
RSV+BALF	33
RSV+MP	31
NI+FBS	31
NI+HP	22
RSV+FBS	25
RSV+HP	26
RSV	33
HSV	19
RSV+jHP	14

Supplementary Figure 3. Correlation matrices for each of the PCAs presented in Figure 1. (**a-c**) Spearman correlation coefficients between each of the samples in the PCA based on precursor area of proteins significantly detected (FDR 1%) in all three replicates, (**a**) for samples included in Fig. 1A, (**b**) For samples included in Fig. 1B and (**c**) for samples included in Fig 1C. (**d**) Average coefficients of variation (CV) calculated for replicates in each corona condition based on protein precursor area.

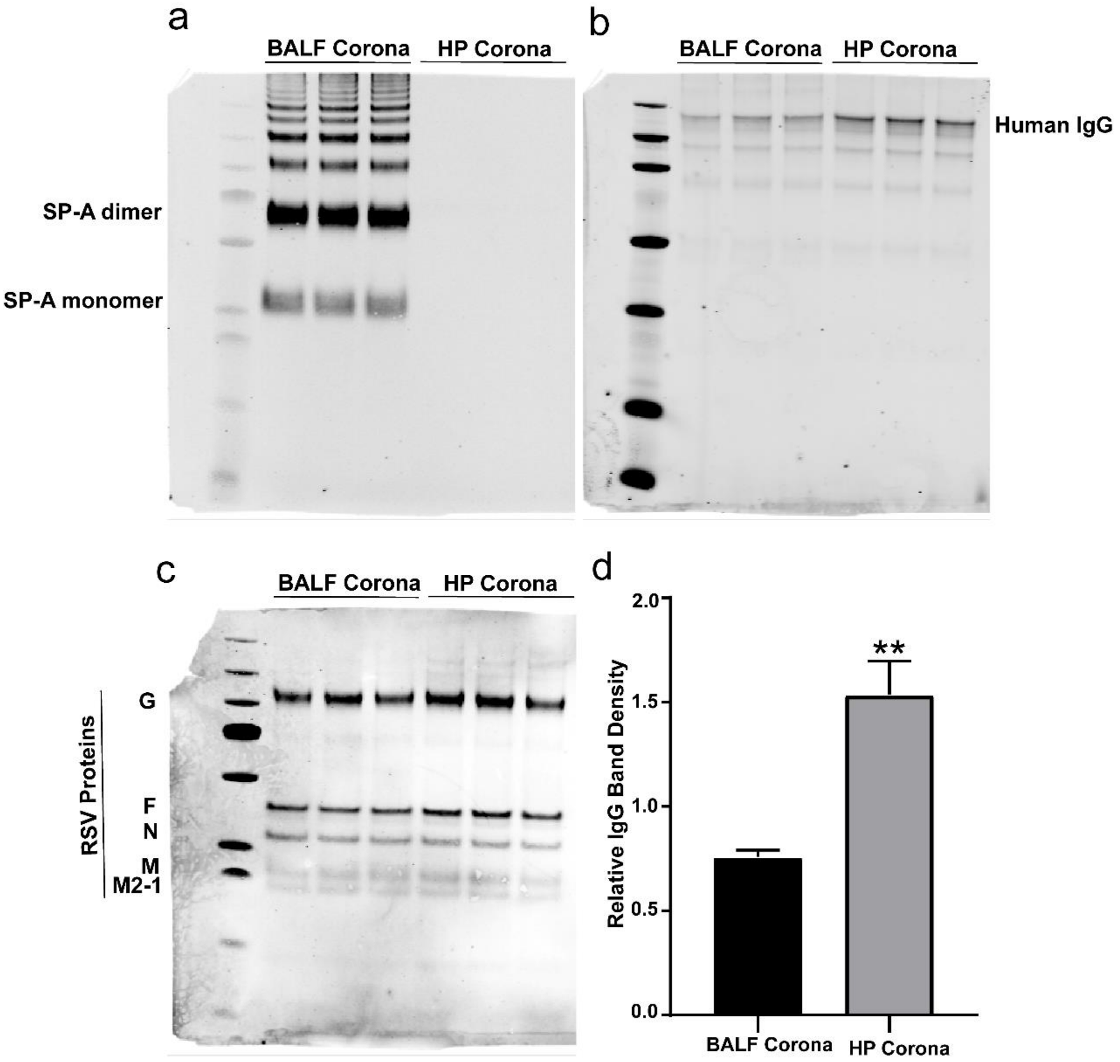

Supplementary Figure 4. Detection of specific corona proteins using western blot. Serum-free produced RSV was incubated with BALF or HP at a protein concentration of 0.1 mg/ml at a ratio of 1:1 v/v for 1h at 37 °C then harvested and washed in a procedure similar to the corona proteomic analysis. (**a**) Bands detected using anti SP-A antibody appear only in the BALF corona samples showing SP-A bands together with higher molecular weight SP-A containing complexes. No SP-A bands were detected in the HP corona samples. (**b**) Bands detected using anti-human IgG antibody appear in both BALF and HP corona, with higher intensity in the latter. (**c**) RSV viral proteins were used as loading controls. (**d**) IgG band intensity was quantified and normalized to the G protein loading control band intensity using ImageJ software. Data is represented as mean relative intensity ±SEM and significance was calculated using Student's T-test and is indicated by **P <.01.

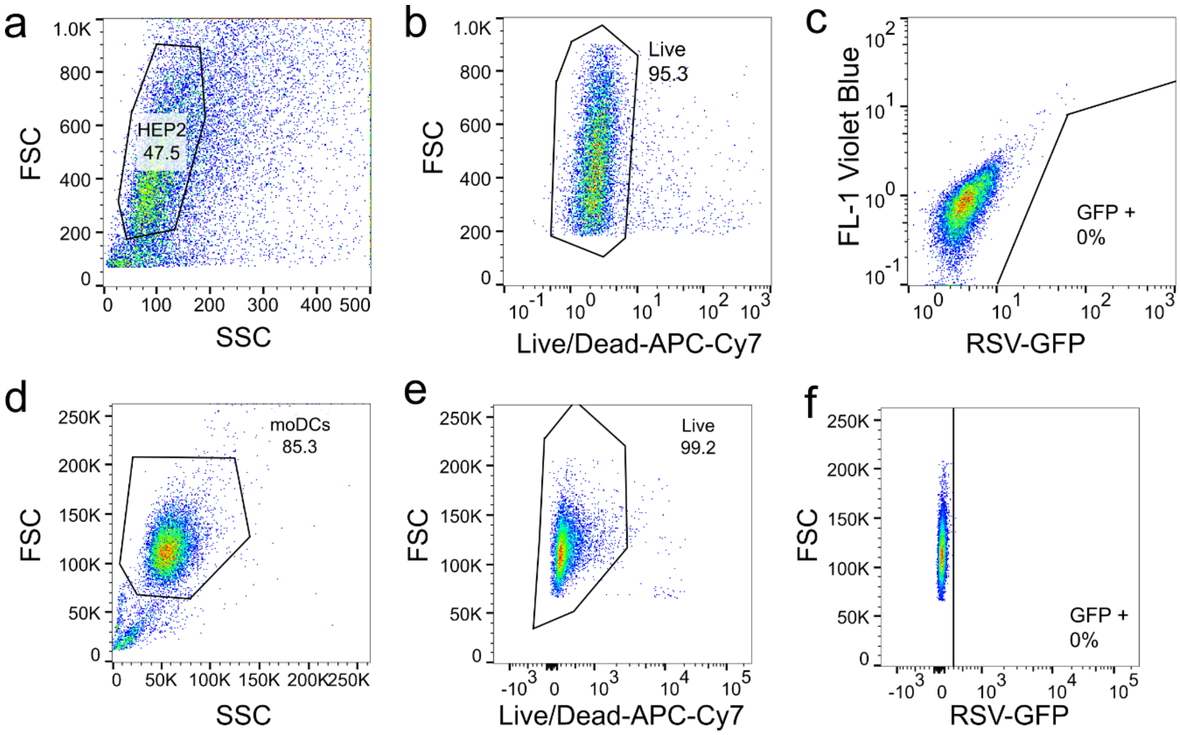

Supplementary Figure 5. Methodology of FACS gating (**a-c**) Uninfected HEp-2 cells and (**d-f**) MoDCs. Cells were first gated based on FSC/SSC (**a** and **d**), followed by gating on living cells (**b** and **e**) and lastly the GFP gate (**c** and **f**). Autofluorescent, non-infected Hep-2 cells were excluded using the FL1 channel.

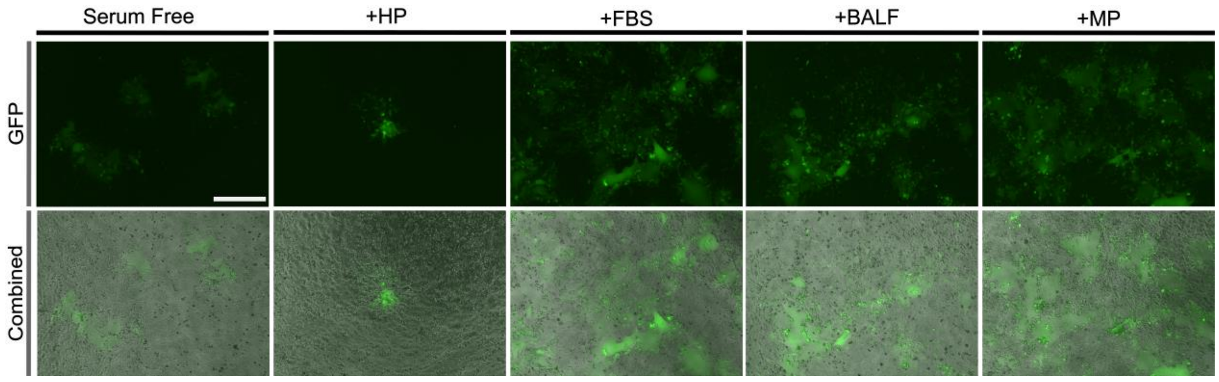

Supplementary Figure 6. Fluorescence microscopy of RSV infectivity. Representative pictures showing infectivity and syncytia formation in in HEp-2 cells using virus stocks in serum-free conditions or with different coronae resulting from pre-incubation with HP, FBS, BALF or MP (n=3). The upper row presents images the fluorescence channel and the lower row represents images of combined fluorescence and phase contrast channels. Bar= 500μM.

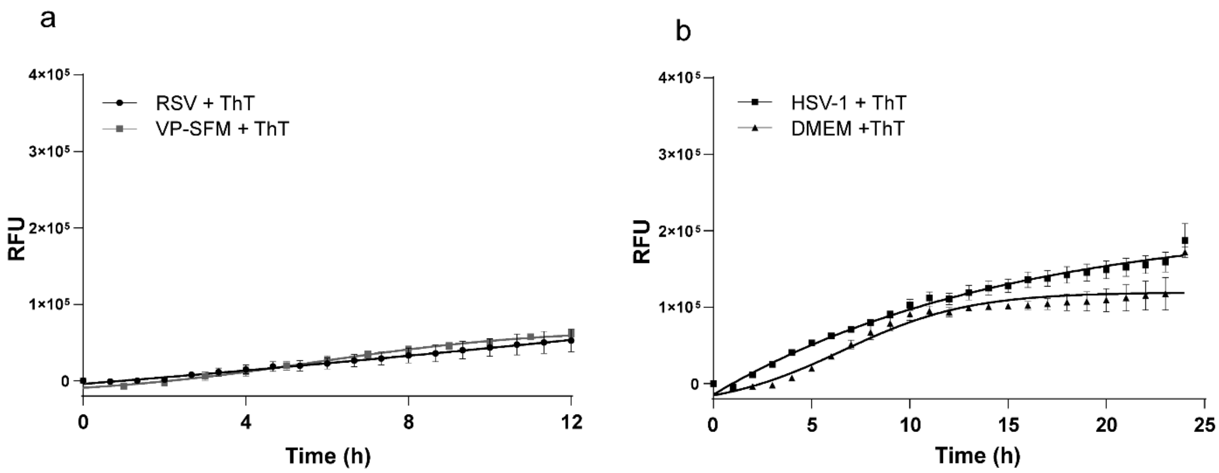

Supplementary Figure 7. Curves representing the background interaction of ThT with the growth media in absence of peptides (**a**) for RSV and (**b**) for HSV-1. Means ±SEM of six replicates from two separate experiments are shown.
