# Supplementary material for: The viral protein corona directs viral pathogenesis and amyloid aggregation
Source: Nat Commun. 2019 May 27;10:2331. doi: 10.1038/s41467-019-10192-2 (PMC6536551; doi:10.1038/s41467-019-10192-2)
Supplement: Supplementary file 4 — Description of Additional Supplementary Files [file 41467_2019_10192_MOESM4_ESM.pdf]

Description of Additional Supplementary Files

File Name: Supplementary Data 1
Description: Total corona proteins detected in all triplicate samples of each biological fluid.

File Name: Supplementary Data 2
Description: Raw proteomics data.
