# Supplementary material for: The viral protein corona directs viral pathogenesis and amyloid aggregation
Source: Nat Commun. 2019 May 27;10:2331. doi: 10.1038/s41467-019-10192-2 (PMC6536551; doi:10.1038/s41467-019-10192-2)
Supplement: Supplementary file 5 — Reporting Summary [file 41467_2019_10192_MOESM5_ESM.pdf]

nature research

Corresponding author(s): *Double-blind peer review submissions: write DBPR and your manuscript number here instead of author names.*

Reporting Summary

Nature Research wishes to improve the reproducibility of the work that we publish. This form provides structure for consistency and transparency in reporting. For further information on Nature Research policies, see Authors & Referees and the Editorial Policy Checklist.

Statistical parameters

When statistical analyses are reported, confirm that the following items are present in the relevant location (e.g. figure legend, table legend, main text, or Methods section).

n/a	Confirmed	
☐	☒	The exact sample size (*n*) for each experimental group/condition, given as a discrete number and unit of measurement
☐	☒	An indication of whether measurements were taken from distinct samples or whether the same sample was measured repeatedly
☐	☒	The statistical test(s) used AND whether they are one- or two-sided<br>*Only common tests should be described solely by name; describe more complex techniques in the Methods section.*
☒	☐	A description of all covariates tested
☐	☒	A description of any assumptions or corrections, such as tests of normality and adjustment for multiple comparisons
☐	☒	A full description of the statistics including central tendency (e.g. means) or other basic estimates (e.g. regression coefficient) AND variation (e.g. standard deviation) or associated estimates of uncertainty (e.g. confidence intervals)
☐	☒	For null hypothesis testing, the test statistic (e.g. *F*, *t*, *r*) with confidence intervals, effect sizes, degrees of freedom and *P* value noted<br>*Give P values as exact values whenever suitable.*
☒	☐	For Bayesian analysis, information on the choice of priors and Markov chain Monte Carlo settings
☒	☐	For hierarchical and complex designs, identification of the appropriate level for tests and full reporting of outcomes
☒	☐	Estimates of effect sizes (e.g. Cohen's *d*, Pearson's *r*), indicating how they were calculated
☐	☒	Clearly defined error bars<br>*State explicitly what error bars represent (e.g. SD, SE, CI)*

Our web collection on statistics for biologists may be useful.

Software and code

Policy information about availability of computer code

Data collection	*Provide a description of all commercial, open source and custom code used to collect the data in this study, specifying the version used OR state that no software was used.*
Data analysis	Quantification of the immunoreactivities was performed using ImageJ 1.50i software (Collins, 2007; Schneider et al., 2012) at a predefined ROI and presented as relative immunoreactive area. For proteomic comparisons, average coefficient of variation (CV) was calculated for each sample based on protein precursor area. Spearman correlation matrices were performed using Morpheus software based on precursor area of proteins significantly detected (FDR 1%) in all three technical replicates in each sample (https://software.broadinstitute.org/morpheus).

For manuscripts utilizing custom algorithms or software that are central to the research but not yet described in published literature, software must be made available to editors/reviewers upon request. We strongly encourage code deposition in a community repository (e.g. GitHub). See the Nature Research guidelines for submitting code & software for further information.

Data

Policy information about availability of data

All manuscripts must include a data availability statement. This statement should provide the following information, where applicable:

- Accession codes, unique identifiers, or web links for publicly available datasets
- A list of figures that have associated raw data
- A description of any restrictions on data availability

The authors declare that the data supporting the findings of this study are available within the paper and its supplementary information files.

Field-specific reporting

Please select the best fit for your research. If you are not sure, read the appropriate sections before making your selection.

☒ Life sciences ☐ Behavioural & social sciences ☐ Ecological, evolutionary & environmental sciences

For a reference copy of the document with all sections, see nature.com/authors/policies/ReportingSummary-flat.pdf

Life sciences study design

All studies must disclose on these points even when the disclosure is negative.

Sample size	Sample size is indicated in each figure legend
Data exclusions	Data was excluded if no RSV infection was obtained in any sample
Replication	Number of replicates are indicated in each figure legend
Randomization	The mice were randomized into treatment groups using GraphPad QuickCalcs.
Blinding	Quantification of the immunoreactivities was carried out blinded to the study groups.

Reporting for specific materials, systems and methods

Materials & experimental systems

n/a	Involved in the study
☐	☒ Unique biological materials
☐	☒ Antibodies
☐	☒ Eukaryotic cell lines
☒	☐ Palaeontology
☐	☒ Animals and other organisms
☐	☒ Human research participants

Methods

n/a	Involved in the study
☒	☐ ChIP-seq
☐	☒ Flow cytometry
☒	☐ MRI-based neuroimaging

Unique biological materials

Policy information about availability of materials

Obtaining unique materials	The use of human BAL samples for the current study was approved by the Regional Committee for Ethical Review in Stockholm (D. No 2016/1985-32). All donors had given oral and written informed consent to participate in the bronchoscopy study, in line with the Helsinki Declaration. The clinical characteristics of the utilized cohort of healthy volunteers have been described in detail elsewhere. The study was approved by the Human Ethics Committee at Huddinge University Hospital, Stockholm (Dnr 75/97). ere (Che KF et al, Am J Resp Crir Care Med 2014). Briefly, healthy subjects of male and female gender were recruited at the Lung Allergy Clinic, Karolinska University Hospital, Solna. These subjects were examined after denying regular tobacco smoking and history of allergy or lung disease during an interview. The final inclusion required that these subjects displayed no signs of pulmonary or disease during clinical examination, spirometry and clinical blood testing including electrolytes, white cell differential counts and C-reactive protein. Bronchoscopy with BAL (5 x 50 ml of sterile and phosphate-buffered saline) was performed according to clinical routine at Karolinska University Hospital, Solna, as previously described (Che KF et al, Am J Resp Crir Care Med 2014). BAL was concentrated using 5 kDa cutoff 4 ml spin concentrator (Agilent Technologies, USA) before infectivity experiments.

jHP was obtained and pooled from at least 3 different infants participating in a prospective cohort of 281 children born into the cohort between 1997 and 2000 in Stockholm, Sweden as has been described in detail elsewhere (Nilsson, C. et al. , J. Allergy Clin. Immunol., 2005) . The study was approved by the Human Ethics Committee at Huddinge University Hospital, Stockholm (Dnr 75/97).

Experiments using buffy coats were carried out in accordance with Swedish guidelines and regulations Ethical permit Dnr 2006/229-31/3. According to regulations in Sweden, experimental in vitro work with cells from buffy coats does not require informed consent.

Monkey plasma was obtained and pooled from at least 3 different Indian rhesus macaques that were RSV seronegative. Ethical permit Dnr N2 / 15, Institutionen för medicin, Karolinska University Hospital, Solna.

Antibodies

Antibodies used

CD86 APC and CD1a Bb510, both from BD biosciences. WO2 from Millipore. anti-Surfactant Protein A antibody [6F10] (ab51891, abcam). Goat anti-human IgG (IRDye 800, red, LI-COR Biosciences) , goat anti-mouse IgG (IRDye 680, green, LI-COR Biosciences). Anti-respiratory syncytial virus antibody (ab20745, abcam). Mouse anti-RSV fusion protein monoclonal antibody (MAB8599, Millipore, USA)

Validation

Describe the validation of each primary antibody for the species and application, noting any validation statements on the manufacturer's website, relevant citations, antibody profiles in online databases, or data provided in the manuscript.

Eukaryotic cell lines

Policy information about cell lines

Cell line source(s)

HEp-2 cells, a human laryngeal carcinoma cell line.
VERO cells

Authentication

Cell lines most commonly used for viral production, not authenticated for this particular study.

Mycoplasma contamination

Cell lines tested negative for mycoplasma contamination

Commonly misidentified lines
(See ICLAC register)

N/A

Animals and other organisms

Policy information about studies involving animals; ARRIVE guidelines recommended for reporting animal research

Laboratory animals

3-month-old female transgenic 5XFAD mice purchased from Jackson Laboratories, Bar Harbor, Maine, US

Wild animals

Provide details on animals observed in or captured in the field; report species, sex and age where possible. Describe how animals were caught and transported and what happened to captive animals after the study (if killed, explain why and describe method; if released, say where and when) OR state that the study did not involve wild animals.

Field-collected samples

For laboratory work with field-collected samples, describe all relevant parameters such as housing, maintenance, temperature, photoperiod and end-of-experiment protocol OR state that the study did not involve samples collected from the field.

Human research participants

Policy information about studies involving human research participants

Population characteristics

The use of human BAL samples for the current study was approved by the Regional Committee for Ethical Review in Stockholm (D. No 2016/1985-32). All donors had given oral and written informed consent to participate in the bronchoscopy study, in line with the Helsinki Declaration. The clinical characteristics of the utilized cohort of healthy volunteers have been described in detail elsewhere (Che KF et al, Am J Resp Crir Care Med 2014). jHP was obtained and pooled from at least 3 different infants participating in a prospective cohort of 281 children born into the cohort between 1997 and 2000 in Stockholm, Sweden as has been described in detail elsewhere (Nilsson, C. et al. , J. Allergy Clin. Immunol., 2005) . The study was approved by the Human Ethics Committee at Huddinge University Hospital, Stockholm (Dnr 75/97).

Recruitment

Healthy subjects of male and female gender were recruited at the Lung Allergy Clinic, Karolinska University Hospital, Solna. These subjects were examined after denying regular tobacco smoking and history of allergy or lung disease during an interview. The final inclusion required that these subjects displayed no signs of pulmonary or disease during clinical examination, spirometry and clinical blood testing including electrolytes, white cell differential counts and C-reactive protein. Bronchoscopy with BAL (5 x 50 ml of sterile and phosphate-buffered saline) was performed according to clinical routine at Karolinska University Hospital, Solna, as previously described (Che KF et al, Am J Resp Crir Care Med 2014).

Flow Cytometry

Plots

Confirm that:

- [x] The axis labels state the marker and fluorochrome used (e.g. CD4-FITC).
- [x] The axis scales are clearly visible. Include numbers along axes only for bottom left plot of group (a 'group' is an analysis of identical markers).
- [x] All plots are contour plots with outliers or pseudocolor plots.
- [x] A numerical value for number of cells or percentage (with statistics) is provided.

Methodology

Sample preparation	Described in materials and methods
Instrument	moDC were acquired on a Fortessa or FACSVERSE (BD Biosciences) and Hep-2 cells on a MACSQuant® Analyser 10 flowcytometer (Miltenyi Biotec, Sweden).
Software	The data was analyzed by FlowJo software (TreeStar) by excluding the dead cells stained with far-red or near IR fluorescent dye and subsequent calculation of GFP positive cells within the viable cell population.
Cell population abundance	No sorting was performed.
Gating strategy	Described in Supp. Fig. 5.<br>Cells were first gated on FSC/SSC, then dead cells were excluded using Live/Dead fixable near-IR dead cell stain kit (ThermoFisher), then GFP positive cells were quantified within the viable cell population.

- [x] Tick this box to confirm that a figure exemplifying the gating strategy is provided in the Supplementary Information.
